# Melatonin inhibits Japanese encephalitis virus replication and neurotoxicity via calcineurin-autophagy pathways

**DOI:** 10.1186/s12868-023-00832-1

**Published:** 2023-11-06

**Authors:** Ji-Hong Moon, Jeong-Min Hong, Jae-Won Seol, Byung-Yong Park, Seong Kug Eo, Sang-Youel Park

**Affiliations:** Biosafety Research Institute, College of Veterinary Medicine, Jeonbuk National University, Gobong ro, Iksan, Jeonbuk 54596 South Korea

**Keywords:** Melatonin, Calcineurin, Japanese encephalitis virus, Autophagy

## Abstract

**Background:**

Japanese encephalitis virus (JEV) is a mosquito-borne flavivirus that has no specific treatment except for supportive medical care. JEV is a neurotropic virus that affects the nervous system and triggers inflammation in the brain.

**Methods:**

Melatonin is used as a sleep-inducing agent in neurophysiology and may serve as a protective agent against neurological and neurodegenerative diseases. Herein, we investigated the effects of melatonin and the critical roles of the serine/threonine protein phosphatase calcineurin during JEV infection in SK-N-SH neuroblastoma cells.

**Results:**

Melatonin treatment decreased JEV replication and JEV-mediated neurotoxicity. Calcineurin activity was increased by JEV infection and inhibited by melatonin treatment. Through calcineurin regulation, melatonin decreased the JEV-mediated neuroinflammatory response and attenuated JEV-induced autophagy.

**Conclusions:**

Calcineurin inactivation has a protective effect in JEV-infected neuronal cells, and melatonin is a novel resource for the development of anti-JEV agents.

**Supplementary Information:**

The online version contains supplementary material available at 10.1186/s12868-023-00832-1.

## Background

Japanese encephalitis (JE) is a mosquito-borne viral encephalitis caused by Japanese encephalitis virus (JEV), which triggers severe inflammation of the brain [1]. JEV is a neurotropic virus that is responsible for frequent epidemics of viral encephalitis in India, China, and Southeast Asia [2]. JEV mainly affects the nervous system, leading to a wide spectrum of clinical symptoms, which constitute a range of conditions from central nervous system (CNS) disorders to febrile syndromes [2]. JE is characterized by extensive inflammation and serious neuronal damage in the CNS [3, 4]. However, the molecular pathogenesis of JEV infection in neurons remains unclear. The identification of cellular molecules related to JEV infection in neurons is crucial for the development of a definitive treatment that inhibits the JEV-mediated neuroinflammatory response and neuronal damage.

Autophagy is an imperative cellular process that maintains cellular homeostasis. During autophagy, autophagic cargo, including long-lived cellular proteins and dysfunctional organelles, is sequestered by autophagosomes and double-membrane vacuoles; subsequently, the cargo is delivered to the lysosome for degradation after autophagosome–lysosome fusion [5]. Various viruses can affect the induction of autophagic flux in infected cells, which is closely associated with the pathogenesis and propagation of these viruses [6–8]. Blocking autophagosomal-lysosomal fusion can also cause accumulation of autophagosomes in virus-infected cells [6, 9, 10]. Functional lysosomes are essential for the maturation of autophagosomes and the degradation of their cargo. Recent studies have proposed that crosstalk between autophagic and apoptotic pathways [9, 11] enables autophagic flux as a response to viral infection, which serves to control apoptosis and virus propagation in infected cells.

Lin et al. suggested that JEV infection causes intracellular calcium (Ca^2+^) overload [12]. Mitochondrial depolarization causes a sustained cytosolic increase in Ca^2+^ level, which activates the cytosolic serine/threonine phosphatase calcineurin (CaN) [13]. CaN is a calcium-mediated type 2B protein phosphatase and a central regulator of cellular functions [14]. High concentrations of CaN have been observed in the brain, implying that CaN has roles in memory regulation, synaptic plasticity, and neuronal disorders [15]. CaN also plays crucial roles in neurons, particularly in apoptosis and the proinflammatory response [16, 17]. Several studies have suggested that an increase in intracellular Ca^2+^ level promotes autophagic flux via various molecular pathways, including CaMKK, AMPK, and mTOR pathways [18–20]. In recent studies, FK506, a CaN inhibitor, has been found to decrease the risk of neurodegenerative disorders, such as Alzheimer’s, Huntington’s, and prion diseases [21–24].

Melatonin is a hormone involved in endogenous defense mechanisms. The neuroprotective effects of melatonin, including its antioxidant and anti-inflammatory effects, antagonize the effects of CNS disorders, such as ischemic brain injury and Parkinson’s disease [25–27]. Melatonin is also known to induce autophagy [28–31]. Previous studies have also suggested that melatonin has a protective effect against the Dengue virus [32] and West Nile virus [33]. However, the effect and mechanism of melatonin against JE have not been investigated, and the precise autophagic regulation in JEV replication remains unclear.

This study aimed to examine the effects of JEV infection on neuronal cell death by assessing autophagy impairment and CaN activation.

## Results

### Melatonin inhibits JEV propagation and cell death in human SK-N-SH neuroblastoma cells

**Fig. 1:**
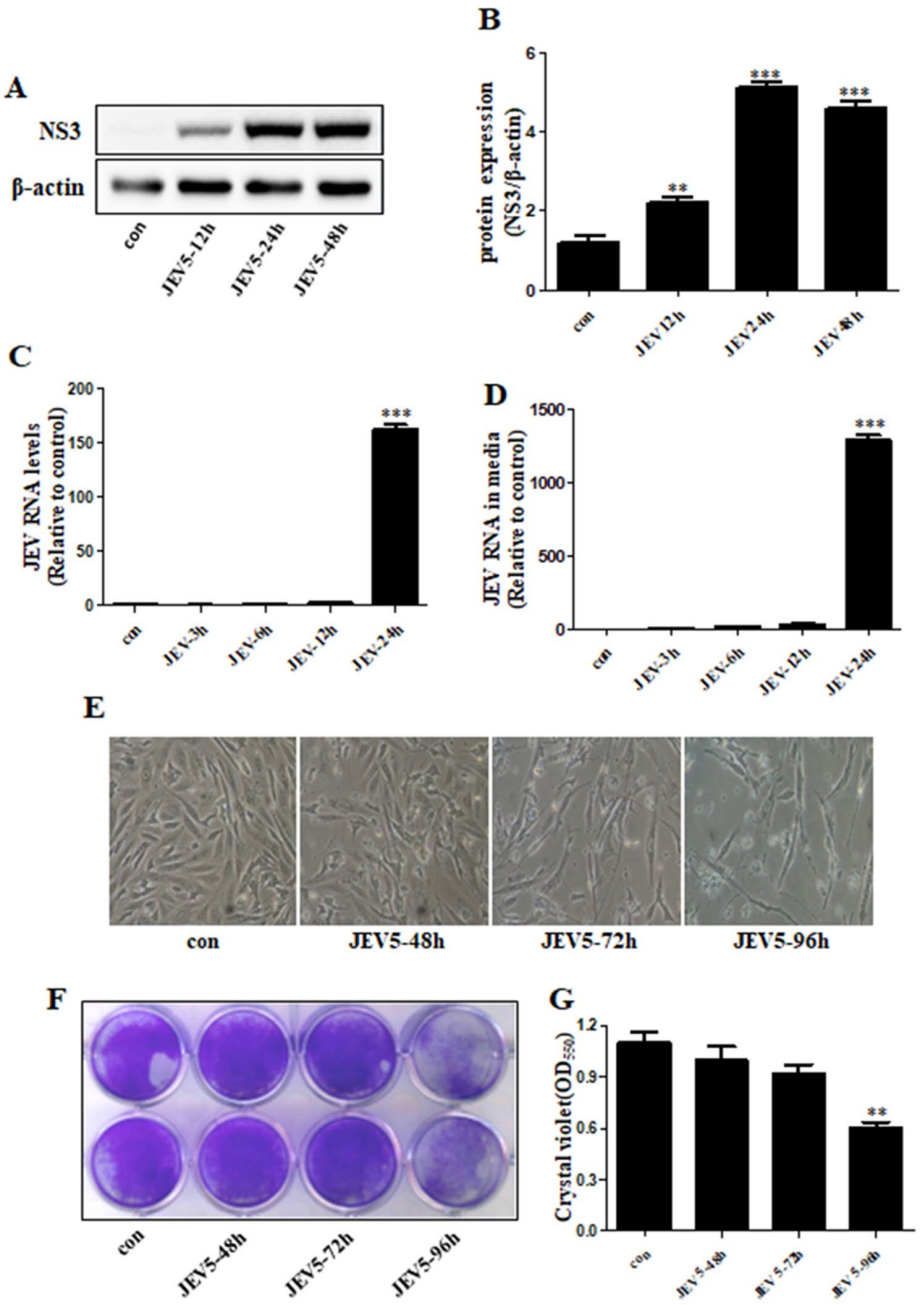
**JEV replication and death of neuronal cells** SK-N-SH cells were infected with JEV at 5 MOI. (**A**) The protein levels of JEV NS3 were checked in control and JEV-infected cells. (**B**) Bar graph showing the average NS3 protein levels normalized to β-actin. (**C**, **D**) JEV RNA levels in cells and culture media indicated by PCR. (**E**) Cells were photographed under the microscope (100x). (**F**) Viable cells were assayed with crystal violet. (**G**) Graphical data showing the OD550 nm from 1% SDS elution with crystal violet stain. Values represent the mean ± SEM (n = 5 [B], 10 [**C**, **D**, **G**]). **p < 0.01, ***p < 0.001 vs. control

SK-N-SH cells were used to investigate neuroprotective activity in vitro because of their neuron-like behavior [34–36]. To examine the susceptibility of SK-N-SH cells to JEV infection and virus replication, SK-N-SH cells were infected with JEV at an MOI of five. Western blot (Fig. 1A, B) and real-time PCR (Fig. 1C) analyses demonstrated that JEV infection increased nonstructural protein 3 (NS3) and JEV RNA levels at 24 h. Using real-time PCR, we identified JEV RNA propagation in culture media (Fig. 1D). JEV infection significantly increased neuronal cell death in a time-dependent manner compared to that in the control group (Fig. 1E); additionally, JEV infection increased cell death to ~ 55% according to the results of photomicrography and crystal violet assay (Fig. 1F, G).

**Fig. 2:**
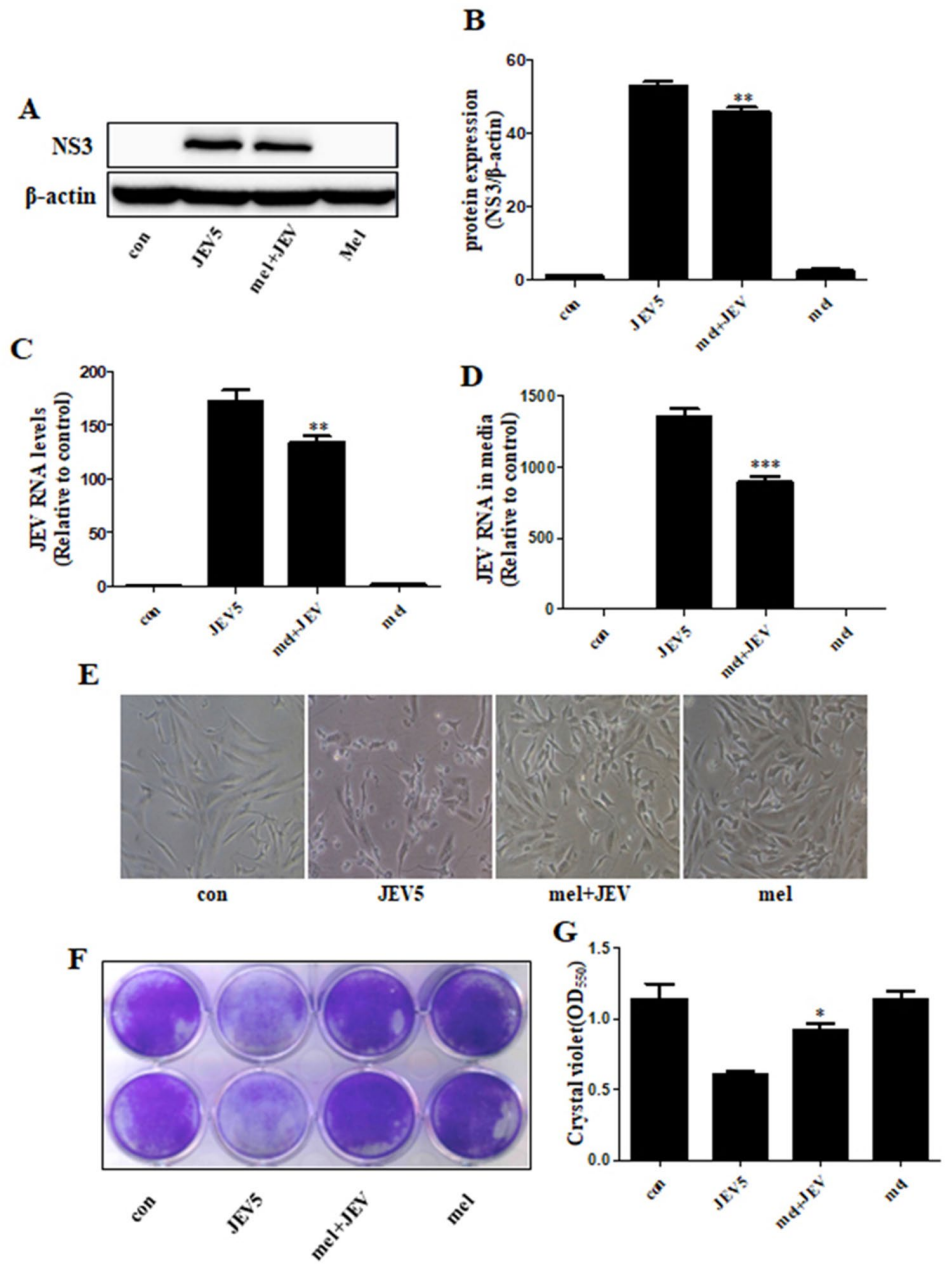
**Melatonin attenuated JEV replication and neuronal cell death** SK-N-SH cells were pretreated with melatonin (100 µM) for 6 h, and then infected with JEV at an MOI of 5.0 for 1 h. Cells were collected after incubation with fresh media for 24 h. (**A**) The protein levels of JEV NS3 and phospho-NFκB were assayed via western blot. (**B**) Bar graph showing the average NS3 protein levels. (**C**, **D**) JEV RNA levels in cells and culture media indicated by PCR. (**E**) Cells were photographed under light microscopy (100x). (**F**) Viable cells were stained with crystal violet. SK-N-SH cells were pretreated with melatonin (100 µM) for 6 h, and then infected with JEV at an MOI of 5.0 for 1 h. Cells were collected after incubation with fresh media for 96 h. (**G**) Graphical data showing the OD550 nm from 1% SDS elution with crystal violet stain. Values represent the mean ± SEM (n = 5 [B], 10 [C, D, G]). *p < 0.05, **p < 0.01, ***p < 0.001 vs. JEV

Next, western blot and real-time PCR analyses were performed to confirm whether melatonin inhibits JEV production. The results showed that melatonin treatment of SK-N-SH cells decreased the intracellular viral protein expression of NS3, phosphorylation of NF-κB (Fig. 2A, B), and RNA level of JEV in the cells and media (Fig. 2C, D). Moreover, the photomicrographs indicated that melatonin reduced JEV-induced neuronal cell death (Fig. 2E), while the crystal violet assay showed that melatonin decreased the cell death of JEV-infected cells (Fig. 2F, G). Our data suggest that melatonin is an antiviral agent against JEV.

### Melatonin attenuates the JEV-mediated neuroinflammatory response

**Fig. 3:**
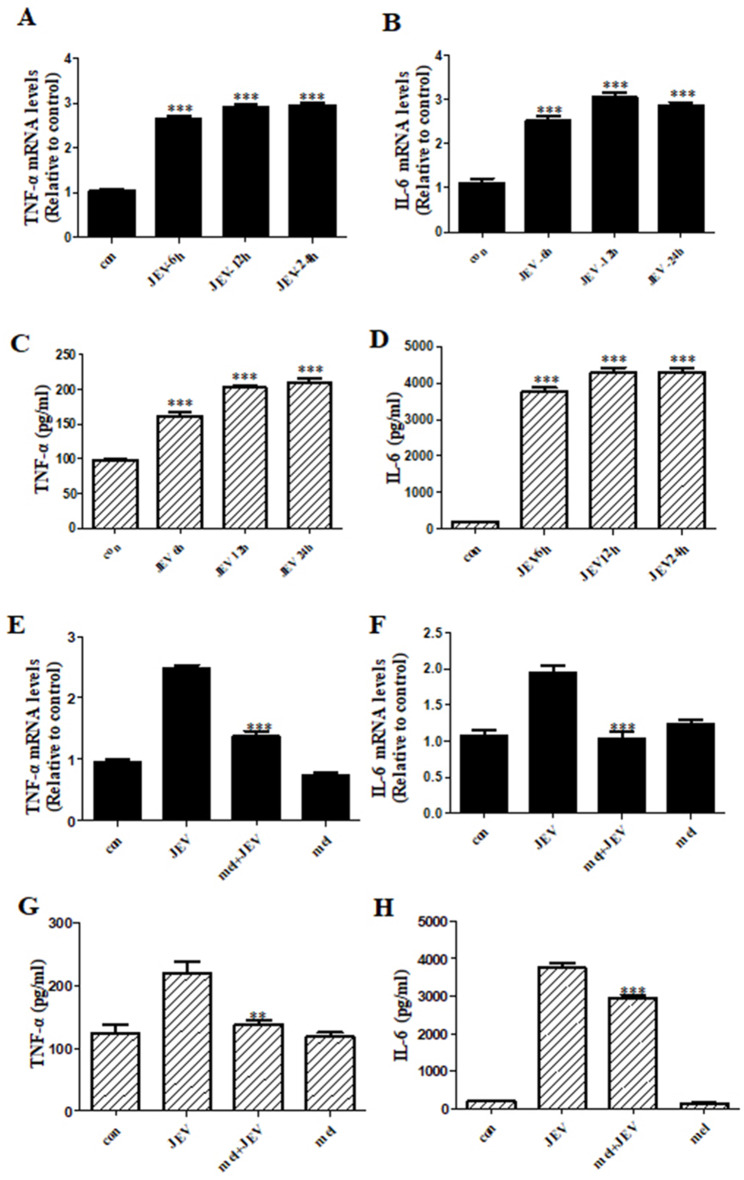
**Melatonin inhibited JEV-mediated neuroinflammatory response** SK-N-SH cells were subjected to infection and melatonin treatment as described in Fig. 2. The mRNA (**A**, **B**, **E**, **F**) and protein (**C**, **D**, **G**, **H**) levels of TNF-α and IL-6 were measured via ELISA and quantitative real-time PCR, respectively. (**A**, **B**, **E**, **F**) The expression levels of TNF-α and IL-6 cytokines were evaluated by normalizing to those of GAPDH; the graph indicates the relative quantification of gene expression normalized to control samples. (**C, D, G, H**) The data show the mean concentrations (pg/ml) of TNF- α and IL-6 secreted in the supernatant. Values represent the mean ± SEM (n = 10). **p < 0.01, ***p < 0.001 vs. control (**A, B, C, D**) or JEV (**E, F, G, H**)

Proinflammatory cytokines, such as TNF-α and IL-6, play a crucial role in mediating the neuroinflammatory response and neurotoxicity in various neurodegenerative diseases [37–39]. Therefore, we next examined the influence of JEV infection on TNF-α and IL-6 levels using qPCR and ELISA. Both TNF-α and IL-6 mRNA expression was significantly increased at 6, 12, and 24 h p.i. (Fig. 3A, B). The secreted pro-inflammatory cytokines were examined in the culture media of SK-N-SH cells using ELISA. In accordance with the mRNA data, the release of TNF-α and IL-6 cytokines gradually increased between 6 and 24 h (Fig. 3C, D). In particular, the expression of the cytokine IL-6 was robustly upregulated after JEV infection. Subsequently, we examined the effect of melatonin on the expression of TNF-α and IL-6 at both the mRNA and protein levels. The results revealed that Japanese encephalitis virus increased TNF-α and IL-6 by more than twofold, and melatonin treatment significantly reduced the mRNA levels of TNF-α and IL-6 (Fig. 3E, F) and repressed the production of inflammatory cytokines (Fig. 3G, H). Taken together, our data demonstrate that melatonin reduces the JEV-mediated production of inflammatory cytokines.

### Melatonin-induced inhibition of CaN decreases the JEV-induced neuroinflammatory response and neurotoxicity

**Fig. 4:**
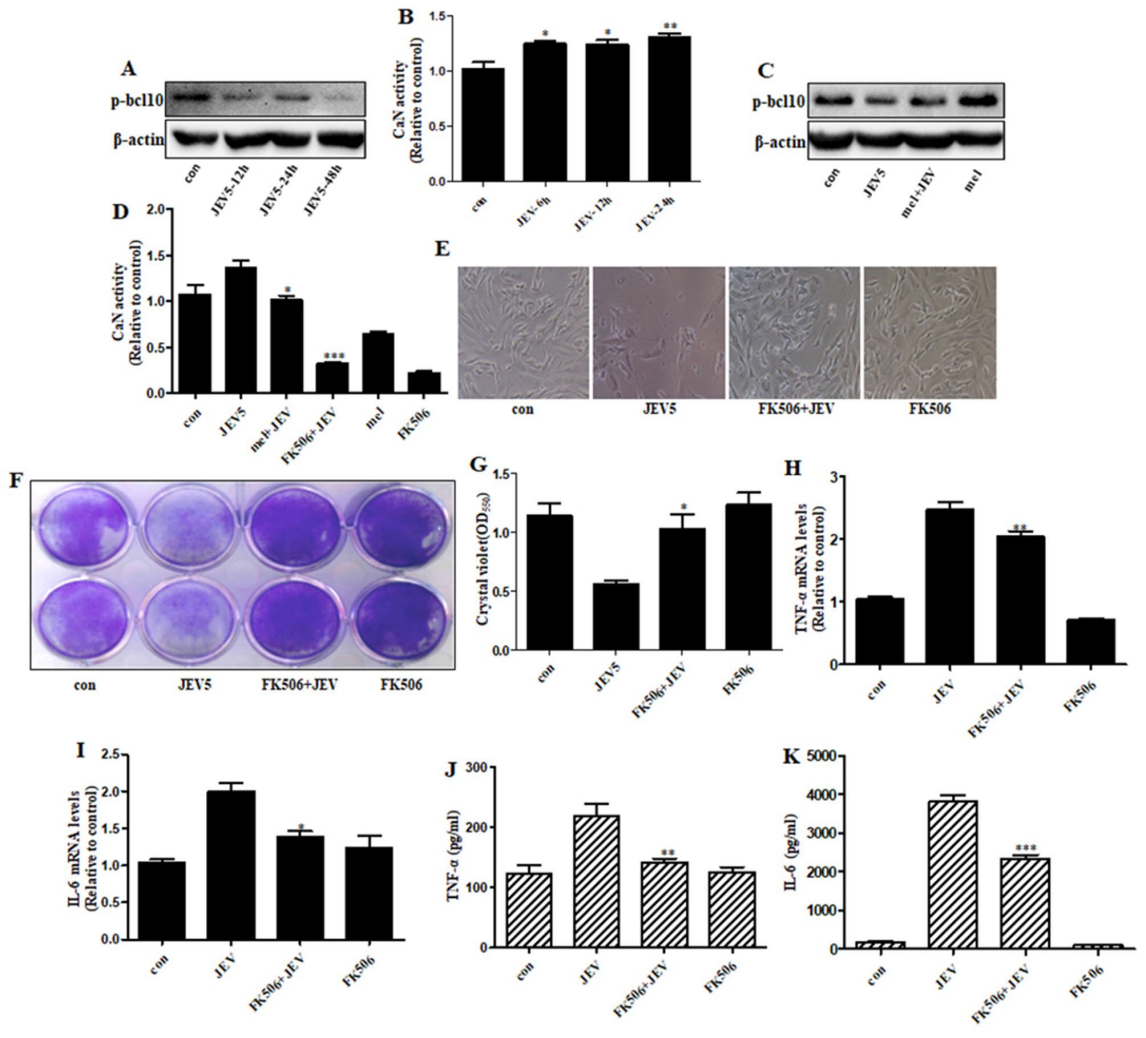
**Melatonin protected JEV-mediated neurotoxicity via inhibition of CaN** (**A**) The protein levels of phospho-bcl10 were examined via western blot. (**B**) CaN activity was measured using a CaN activity assay. (**C**) SK-N-SH cells were previously treated with melatonin (100 µM) for 6 h, and then infected with JEV at an MOI of 5.0 for 1 h. Cells were collected after incubation with fresh media for 24 h. The protein levels of phospho-bcl10 were examined via western blot using the indicated primary antibodies. (**D**) CaN activity was measured by CaN activity assay. (**E**) Cells were observed under light microscopy (100x). (**F**) Viable cells were measured by crystal violet. SK-N-SH cells were pretreated with FK506 (10 µM) for 1 h, and then infected with JEV at an MOI of 5.0 for 1 h. Cells were collected after incubation with fresh media for 96 h. (**G**) Graphical data showing the OD550 nm from 1% SDS elution with crystal violet stain. (**H**) SK-N-SH cells were subjected to infection and treatment with FK506. Cells were pretreated with FK506 (10 µM) for 1 h, and then subjected to JEV infection at an MOI of 5.0 for 1 h. Cells were collected after incubation with fresh media for 24 h. The mRNA and protein levels of TNF-α and IL-6 were measured via quantitative real-time PCR (**H, I**) and ELISA (**J, K**), respectively. (**H, I**) The expression levels of TNF-α and IL-6 were calculated by normalizing to those of GAPDH; the graph indicates the relative quantification of gene expression normalized to control samples. (**J, K**) The data are expressed as the mean concentration (pg/ml) of TNF- α and IL-6, secreted in the supernatant. Values represent the mean ± SEM (n = 5 (**B, D**), 10 (**G, H, I, J, K**)). *p < 0.05, **p < 0.01, ***p < 0.001 vs. control (**B**) or JEV (**D, G, H, I, J, K**)

Recent studies have suggested that CaN is a significant therapeutic target for the treatment of neurodegenerative diseases [40, 41]. To investigate the effect of JEV infection on CaN alteration, we examined the phosphorylation levels of bcl-10 and CaN, as CaN can dephosphorylate endogenous phosphor-bcl-10 [42]. We observed that JEV infection decreased the protein expression of phospho-bcl-10 and increased CaN activity in a time-dependent manner (Fig. 4A, B), whereas melatonin treatment reversed the JEV-mediated alteration of phospho-bcl-10 and CaN activity. In addition, it was confirmed that FK506 reduced CaN activity and that melatonin also had the same function (Fig. 4C, D). This demonstrated the protective effect of melatonin against JEV-mediated neurotoxicity and CaN alteration. We also investigated whether FK506, a CaN inhibitor, had a protective effect against JEV-induced cell death and the neuroinflammatory response. The results of microscopy (Fig. 4E) and crystal violet assays (Fig. 4F, G) showed that FK506 treatment attenuated JEV-mediated neuronal cell death. We also obtained evidence that FK506 treatment reversed the JEV-mediated upregulation of TNF-α and IL-6 using real-time PCR (Fig. 4H, I) and ELISA (Fig. 4J, K).

### Melatonin attenuated JEV-induced autophagy impairment via CaN alteration

**Fig. 5:**
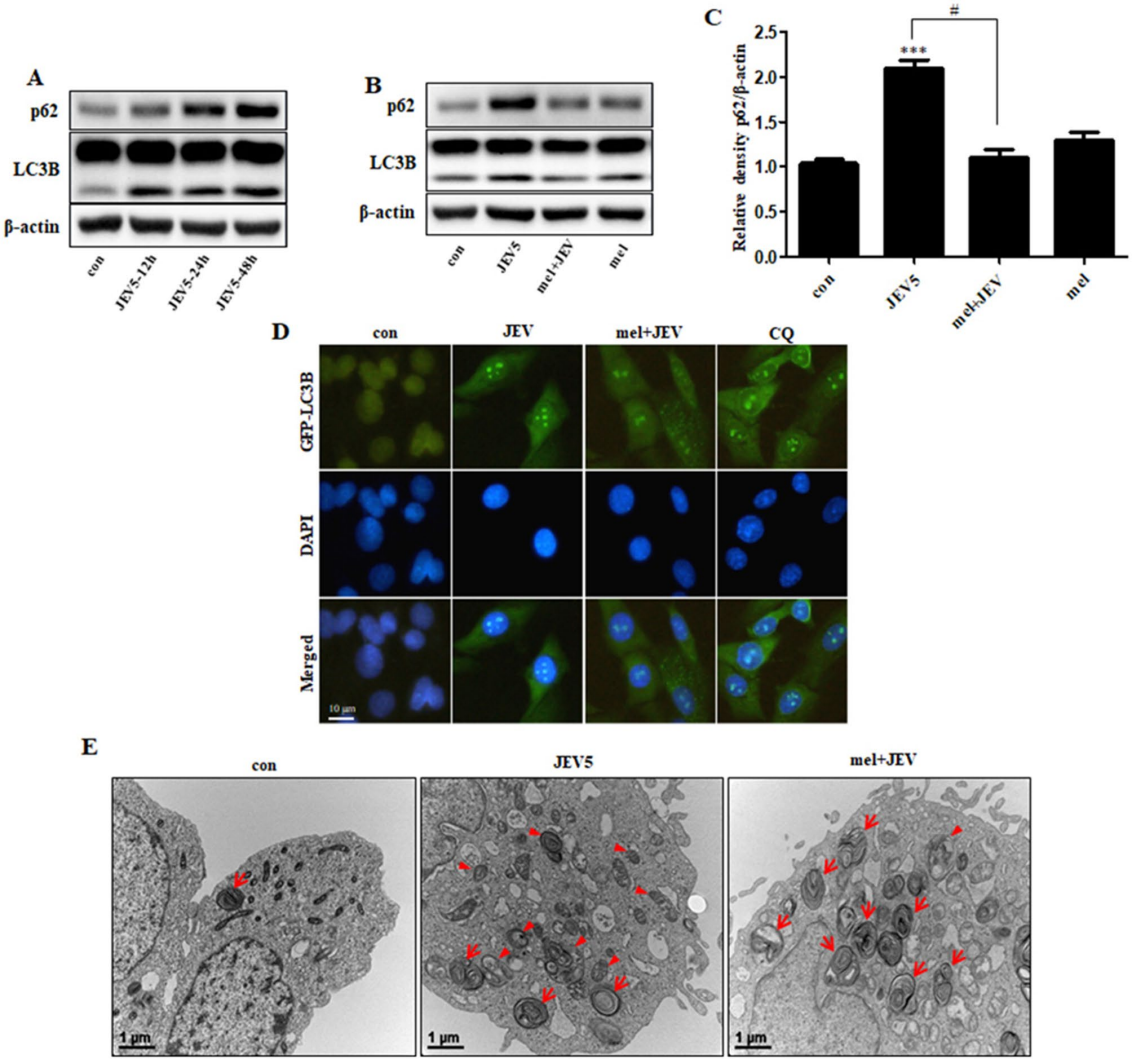
**Melatonin attenuated JEV-induced autophagy impairment** (**A**) The protein levels of SQSTM1/p62 and LC3B were examined. (**B**) SK-N-SH cells were treated with melatonin (100 µM) for 6 h, and then infected with JEV. The protein levels of SQSTM1/p62 and LC3B were analyzed. (**C**) Bar graph representing the average SQSTM1/p62 protein levels. Values represent the mean ± SEM (n = 5). ***p < 0.001 vs. control. # *p* < 0.05 vs. JEV. (**D**) After infection with JEV, the cells were incubated with GFP-LC3B lentivirus at an MOI of 30 for at more than 18 h. Cell nuclei were stained with DAPI (blue) and analyzed using confocal microscopy. (**E**) Autophagy flux was examined by TEM. Arrowheads designate autophagosomes and arrows designate autolysosomes

Previous studies have indicated that JEV infection activates autophagy in neurons [4, 43, 44]. To observe the role of JEV in the induction of autophagy, we monitored the levels of LC3-II and SQSTM1/p62 (p62) following JEV infection. The results revealed that JEV infection increased LC3-II levels, which was indicative of induction of autophagy (Fig. 5A). JEV infection also markedly increased the expression of SQSTM1/p62, a selective autophagy substrate that forms a scaffold for protein aggregates and causes autophagic degradation, which was indicative of autophagosomal–lysosomal blockade in JEV-infected cells.

We next investigated the relationship among melatonin, CaN, and autophagy following JEV infection. Melatonin treatment led to the degradation of SQSTM1/p62, indicating autophagosome turnover by lysosomal proteolysis (Fig. 5B, C). We also observed that JEV infection increased GFP-LC3 fluorescence, while melatonin slightly decreased the numbers of GFP-LC3 puncta because of lysosomal degradation (Fig. 5D). Moreover, the formation of several vesicles, including double-membraned autophagosomes, was induced by JEV infection, indicating inhibition of lysosomal degradation (Fig. 5E). Melatonin treatment induces autophagic degradation through single-membrane autolysosomes.

**Fig. 6:**
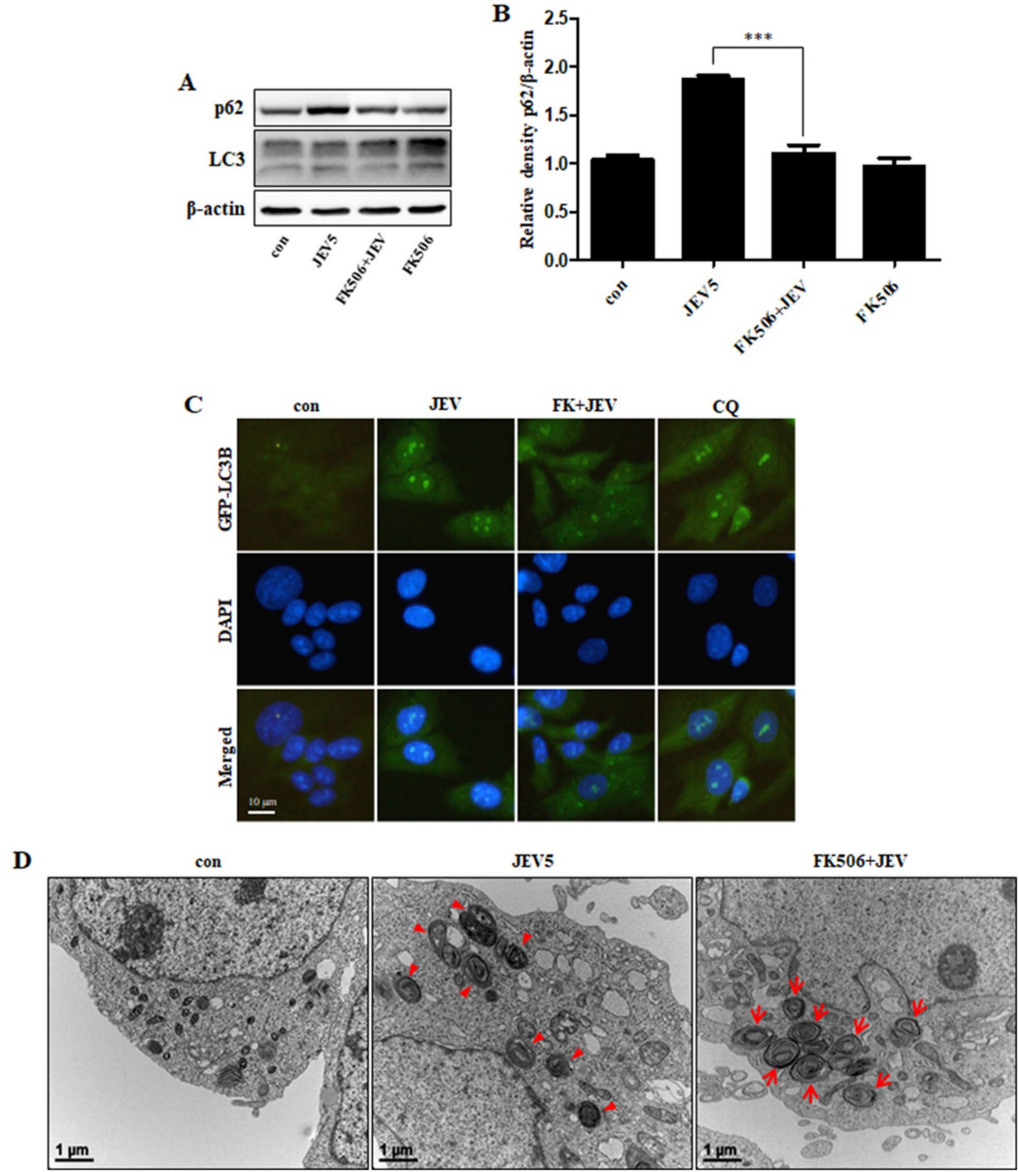
**CaN regulated JEV-induced autophagy impairment** (**A**) SK-N-SH cells were treated with FK506 (10 µM) for 1 h, and then infected with JEV. The protein levels of SQSTM1/p62 and LC3B were examined. (**B**) Bar graph representing the average SQSTM1/p62 protein levels. Values represent the mean ± SEM (n = 5). ***p < 0.001 vs. JEV. (**C**) After infection with JEV, the cells were incubated with GFP-LC3B lentivirus at an MOI of 30 for more than 18 h. Cell nuclei were stained with DAPI (blue) and analyzed by confocal microscopy. (**D**) Autophagy flux was examined by TEM. Arrowheads designate autophagosomes and arrow designate autolysosomes

We next employed the CaN inhibitor FK506 to investigate whether melatonin induced autophagic degradation through CaN. The results revealed that FK506 reversed the increase in SQSTM1/p62 in JEV-infected cells (Fig. 6A, B), slightly decreased the number of GFP-LC3 puncta because of lysosomal degradation (Fig. 6C), and improved autophagic degradation by creating autolysosomes (Fig. 6D). These results indicate that melatonin induces autophagic degradation through CaN alterations in JEV-infected cells.

## Methods

### Cells and viruses

The human neuroblastoma cell line SK-N-SH was purchased from the American Type Culture Collection (ATCC, Rockville, MD, USA) and propagated as previously described [45]. The cells were cultured in minimum essential medium (MEM, HyClone Laboratories, Logan, UT, USA) containing 10% fetal bovine serum (Invitrogen-GIBCO, Grand Island, NY, USA) and gentamycin (0.1 mg/mL) in a humidified incubator maintained at 37 °C under 5% ${CO}_{2}$. SK-N-SH cells were grown to 90% confluence in six-well plates (4 × 10^5^ cells/well) before being infected with JEV (2 × 10^6^ plaque-forming units [PFU]) at a multiplicity of infection (MOI) of five. The log value of the virus titer at 5 MOI was approximately 6.301. The JEV Beijing-1 strain was cultured in a mosquito cell line, C6/36, in Dulbecco’s modified Eagle medium (DMEM), as described previously [46]. Virus stocks were titrated using traditional plaque-forming assays and stored in aliquots at − 80 °C until use. Briefly, the viruses were adsorbed for 1 h at 37 °C. After 1 h, nonbinding viruses were removed by washing twice with phosphate-buffered saline, and the cells were further incubated with fresh media.

### Chemical treatment

Stock solutions of melatonin (100 mM in ethanol) and FK506 (10 mM in dimethyl sulfoxide (DMSO)) were used.

### Quantitative real-time reverse transcriptase-PCR (qRT‒PCR) analysis

All RNA was collected using a Total RNA Extraction Kit. cDNA synthesis was performed using a TOPscript™ cDNA Synthesis Kit. Quantitative real-time polymerase chain reaction (PCR) was conducted using 1 µL of gene-specific primers and SYBR Green PreMIX. The following primers were used: JEV, forward, 5′CCC TCA GAA CCG TCT CGG AA 3′ and reverse, 5′ CTA TTC CCA GGT GTC AAT ATG CTG T 3′; TNF-α, forward, 5′TCT CCT TCC TGA TCG TGG C′ and reverse, 5′ GGT TCA GCC ACT GGA GCT 3′; and IL-6, forward, 5′ AAA TTC GGT ACA TCC TCG AC 3′ and reverse, 5′ CAG GAA CTG GAT CAG GAC TT 3′. All qPCRs were performed using a CFX96 real-time PCR system (Bio-Rad).

### Enzyme-linked immunosorbent assay (ELISA)

TNF-α and IL-6 protein levels in culture media were assayed using immunoenzymatic kits (TNF-α and IL-6 LEGEND MAX^™^ ELISA kits, BioLegend, San Diego, CA) according to the manufacturer’s protocol; subsequently, they were analyzed using a SpectraMax M2 micro reader (Molecular Devices).

### Crystal violet assay

The cell morphology was evaluated using an inverted microscope. Cells were prepared for cell viability analysis using crystal violet (C0775; Sigma‒Aldrich) staining, as previously reported [47]. Briefly, cells were stained for 10 min at RT with crystal violet solution (0.5% crystal violet in 30% ethanol and 3% formaldehyde), washed five times with water, and dried. After that, the cells were lysed with 1% SDS (sodium dodecyl sulfate), and the absorbance was measured at 550 nm.

### Calcineurin activity assay

CaN activity was measured using a calcineurin cellular activity assay kit (no. BML-AK816-0001; Enzo Life Sciences), as previously reported [48]. The reaction was estimated by measuring the absorbance (OD) of malachite green in the extract at 620 nm using a SpectraMax M2 microplate reader (Molecular Devices).

### Western blot analysis

Cells were lysed in lysis buffer containing radioimmunoprecipitation assay (RIPA) buffer, phenylmethanesulfonyl fluoride (PMSF), sodium orthovanadate (Na_3_VO_4_), and a protease inhibitor mixture. Equal quantities of proteins were electrophoretically separated on 10% sodium dodecyl sulfate‒polyacrylamide gels (SDS‒PAGE) and transferred to nitrocellulose membranes (Pall Corp., USA). The membranes were incubated with 5% skim milk in Tris-buffered saline (TBS) buffer with 0.1% Tween-20 for 1 h at room temperature (RT). Subsequently, the membranes were incubated with the indicated primary antibodies for 2 h and with secondary antibodies conjugated to horseradish peroxidase (HRP) for 1 h. Immunoreactive bands were developed using enhanced chemiluminescence reagents (LF-QC0103, AbFrontier Inc.). We used primary antibodies against NS3 (GTX125868, GeneTex), phospho-Bcl10 (sc-81,484, Santa Cruz Biotechnology), phospho-NFκB, LC3B, P62 (#3033, #4108, and #5114, Cell Signaling Technology), and β-actin (A5441, Sigma–Aldrich).

### BacMam Transduction

SK-N-SH cells were incubated with GFP-tagged LC3B reagent for 24 h and then treated with melatonin, FK506, CQ (chloroquine), or JEV, as described previously [49]. LC3B-FP and LC3B (G120A)-FP viral vectors (MOI = 30) were transduced into cells, enabling the expression of fluorescent LC3B protein. Autophagosome dynamics were then monitored using an inverted fluorescence microscope.

### TEM (transmission electron microscopy) analysis

SK-N-SH cells were incubated with melatonin, FK506, or JEV; subsequently, TEM analysis was performed as previously described [45]. Briefly, SK-N-SH cells were fixed with 2% paraformaldehyde and 2% glutaraldehyde in 0.05 M sodium cacodylate, pH 7.2. The cells were postfixed with 1% osmium tetroxide for further fixation. Specimens were dehydrated in graded ethanol, the ethanol was changed to propylene oxide, and the specimens were then embedded in epoxy resin. Images were analyzed using a Hitachi H7650 electron microscope (Hitachi, Ltd., Tokyo, Japan; magnification, 10,000×) at the Center for University-Wide Research Facilities, Jeonbuk National University.

### Statistical analysis

The results are shown as the mean ± standard error from at least three independent experiments. Data among multiple independent groups were compared using one-way analysis of variance with Tukey’s post hoc test. All graphical data were analyzed using GraphPad Prism version 5.0. P values < 0.05 were considered to indicate statistical significance.

## Discussion

Herein, we examined the relationship between the cellular autophagy pathway and melatonin-mediated CaN alterations in the context of JEV infection. Autophagy was impaired by JEV infection through CaN upregulation; however, CaN inhibition by melatonin attenuated neuronal cell death through autophagic lysosomal degradation.

Autophagy plays a critical role in the preservation of cellular homeostasis in several types of viral infections [50, 51] but exhibits two opposing roles in viral infection. Herpes simplex virus type 1 represses the autophagic pathway using an advanced mechanism to facilitate its survival [52]; in contrast, hepatitis C virus and poliovirus manipulate autophagy to enhance viral replication [53]. JEV appears to exert both of these opposing effects on autophagy, as JEV has evolved to develop autophagy while suppressing viral replication [4, 43]. Previous studies have suggested that an increase in LC3 is a marker for the induction of autophagy [4, 43, 54]. Here, JEV infection increased SQSTM1/p62 levels, indicating that JEV infection inhibited the interaction between autophagosomes and lysosomes (Fig. 5A). We demonstrated that JEV replication induced autophagy impairment in a human neuroblastoma cell line. Melatonin attenuated autophagy impairment (Fig. 5B). These findings indicate that melatonin, by attenuating autophagy impairment, acts as an antiviral agent in response to JEV infection in neurons.

CaN activates transcription factors of the nuclear factor of activated T cells family, which are the main controllers of the immune response [55, 56]. FK506, an immunosuppressive medicine, is mainly used to lower the risk of rejection in organ transplantation. FK506 binds to the protein FKBP12, which attaches to CaN and inhibits its activity and the resulting immune response [57–59]. Recent studies have suggested a connection between FK506 and anti-neurodegenerative activity, although this is a less-studied biological feature of this drug. FK506 has also been demonstrated to decrease the negative effects that occur in neurodegenerative diseases [22, 23, 60, 61] and is an autophagy inducer [62–64]. Both FK506 and melatonin have similar neuroprotective properties and effects on autophagic pathways, although melatonin augments the immune response [65, 66]. Despite the opposing actions of FK506 and melatonin on the immune system, they may have similar functions in modulating CaN and autophagy in the context of neurodegeneration. Our results also showed that autophagy was activated by FK506 and melatonin treatment in neural cells (Fig. 5B A). These findings suggest that replication of JEV leads to impaired autophagy and is regulated by CaN.

To our knowledge, this is the first study to suggest that melatonin-mediated CaN alteration is partially associated with autophagy and has a neuroprotective effect against the JEV-mediated neuroinflammatory response and neurotoxicity. However, the mechanism underlying melatonin-mediated CaN alteration and autophagy remains unclear. Further studies are needed to examine the melatonin-related mechanisms of the CaN-autophagy pathway in JEV-mediated neurotoxicity in vitro and/or in vivo. In summary, melatonin prevents the JEV-induced neuroinflammatory response and neurotoxicity in neuronal cells.

### Electronic supplementary material

Below is the link to the electronic supplementary material.

Supplementary Material 1

Supplementary Material 2

## Data Availability

The datasets used and/or analyzed during the current study are available from the corresponding author upon reasonable request.

## List of abbreviations

JEV: Japanese encephalitis virus
JE: Japanese encephalitis
CNS: central nervous system
CaN: calcineurin
NS3: nonstructural protein 3
LC3-I/II: microtubule-associated protein 1 A/1B-light chain 3-I/II
p62: SQSTM1/p62
CQ: chloroquine
