# Supplementary figures and images for: Melatonin inhibits Japanese encephalitis virus replication and neurotoxicity via calcineurin-autophagy pathways
Source: BMC Neurosci. 2023 Nov 6;24:59. doi: 10.1186/s12868-023-00832-1 (PMC10629071; doi:10.1186/s12868-023-00832-1)

Fig.1

A

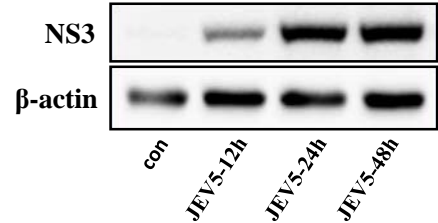

NS3

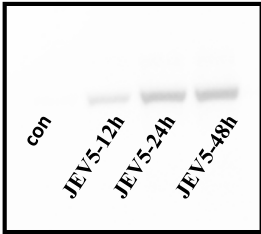

$\beta$ -actin

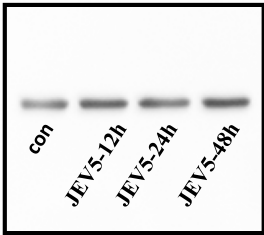

Fig.2 A

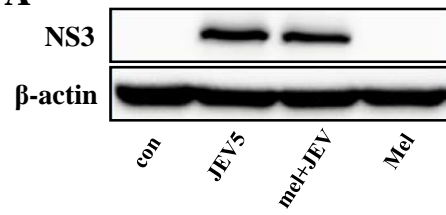

NS3

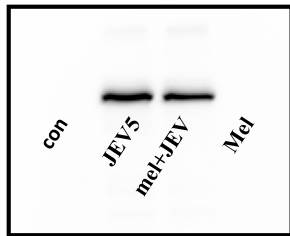

$\beta$ -actin

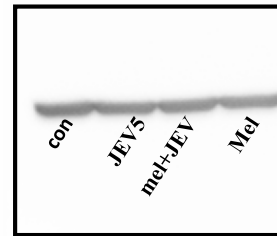

Fig.4

A

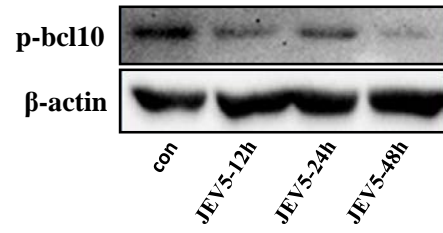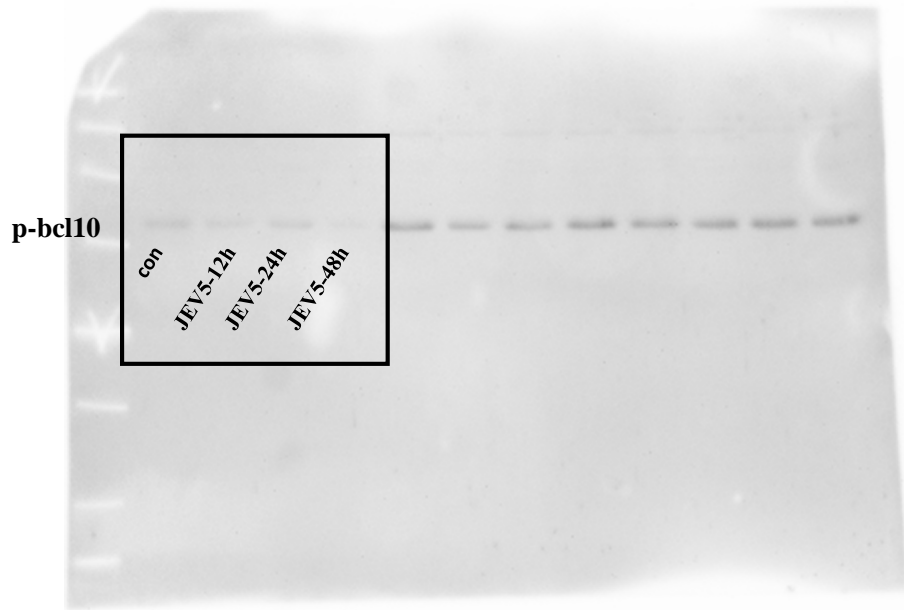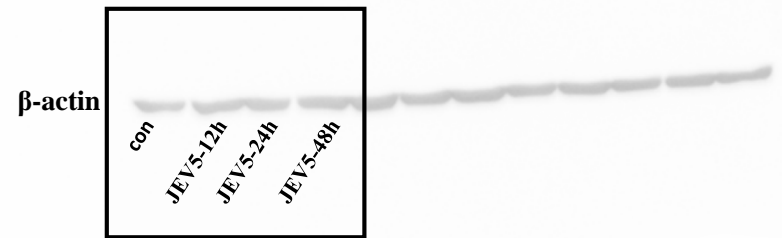

Fig.4 C

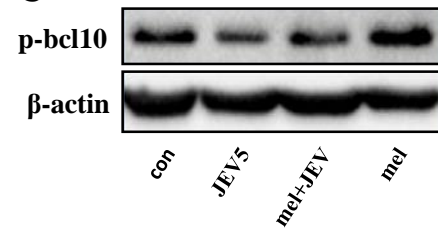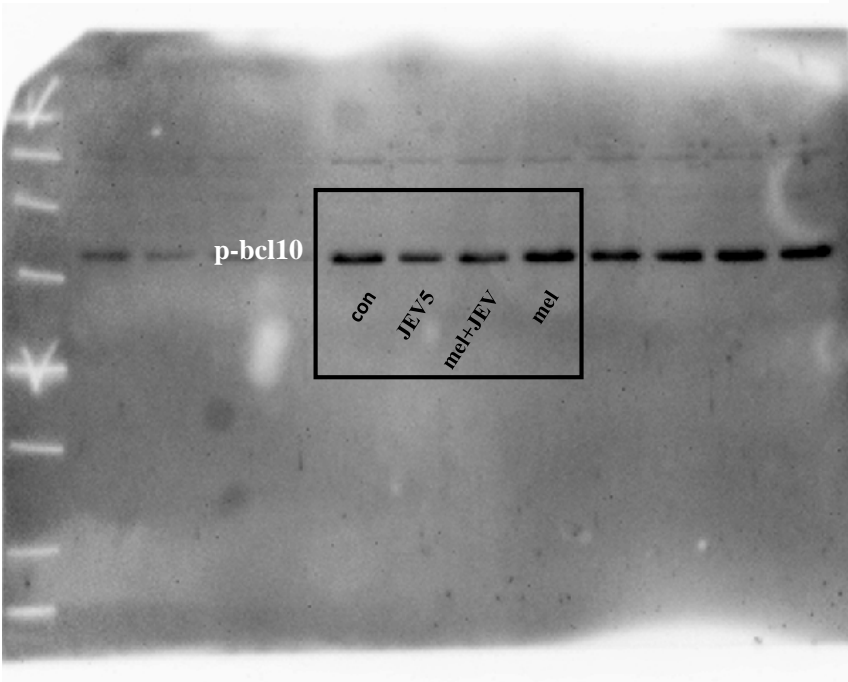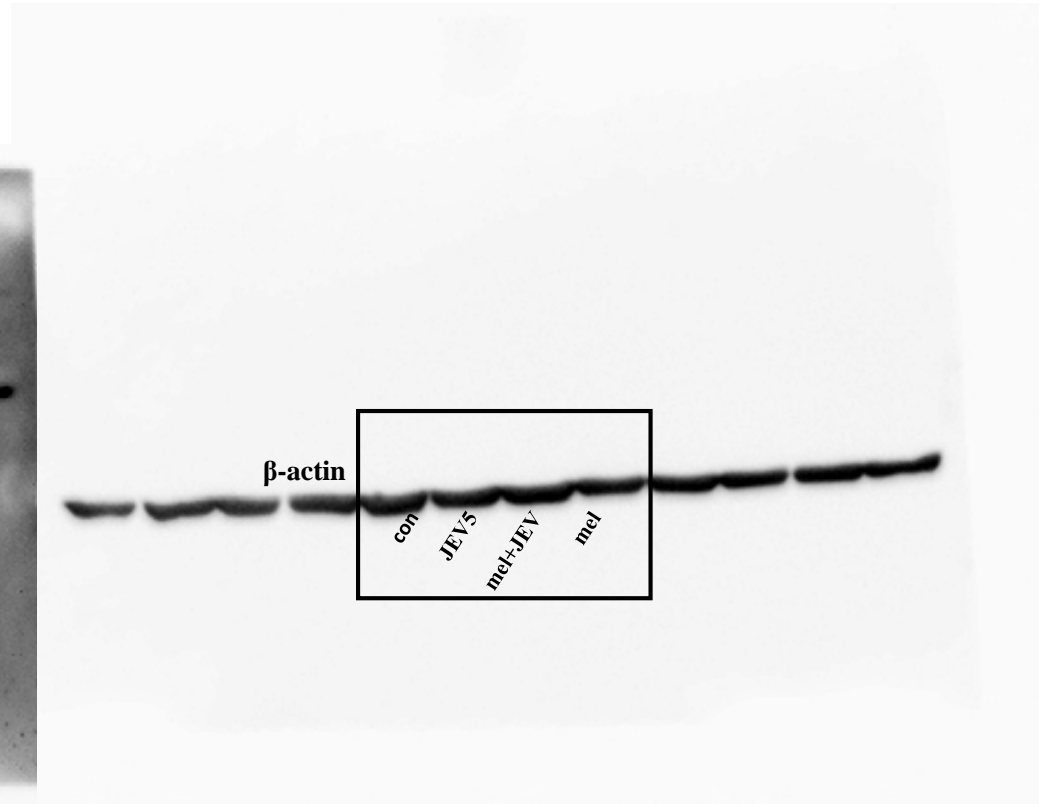

Fig.5

A

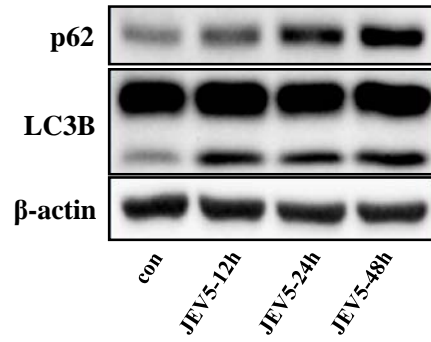

p62

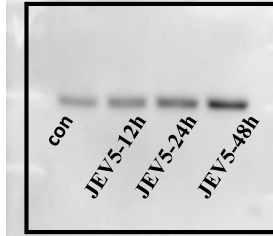

LC3B

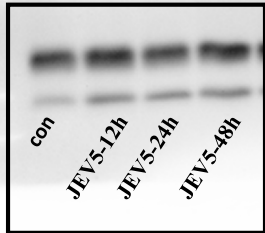

$\beta$ -actin

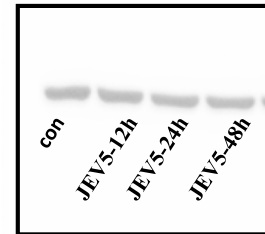

Fig.5

**B**

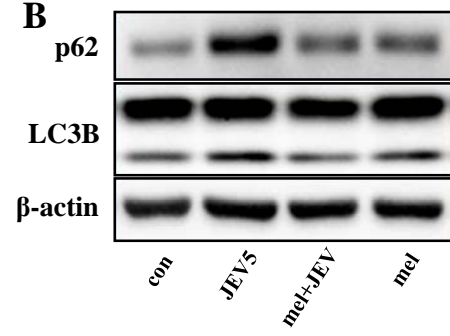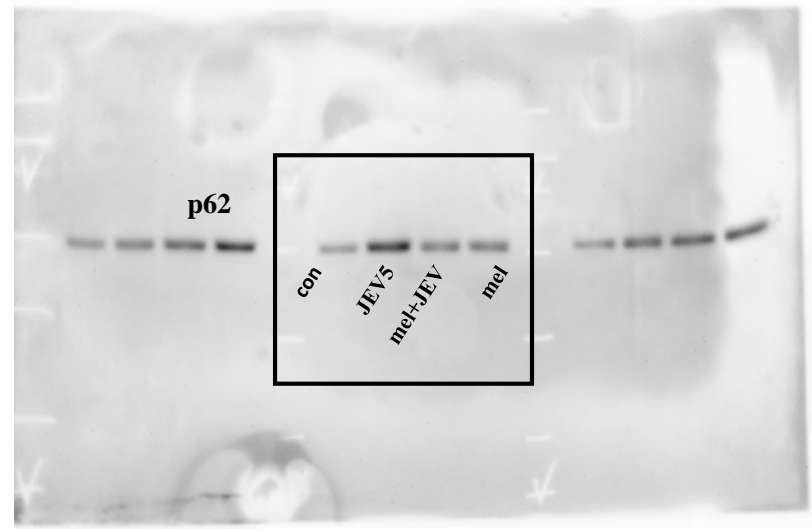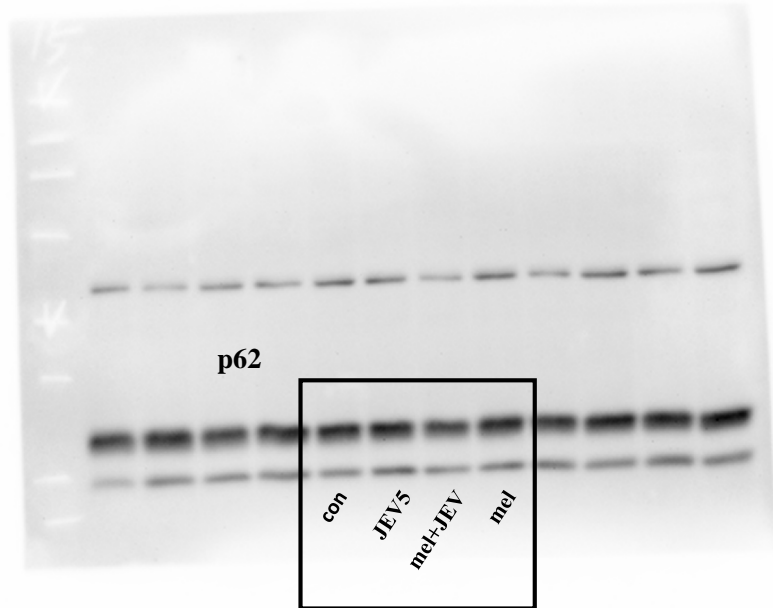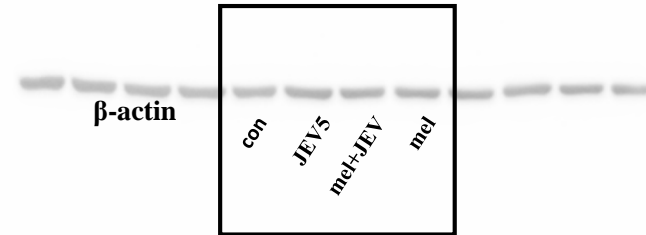

Fig.6

A

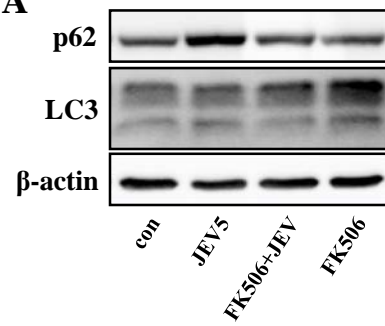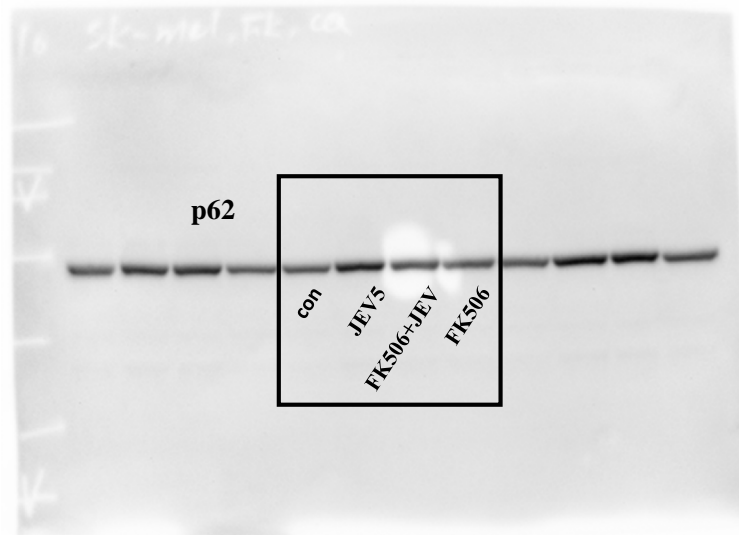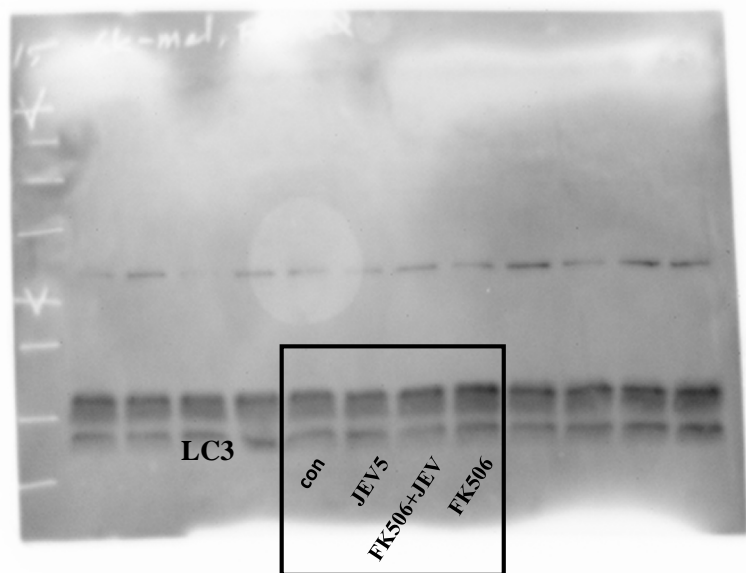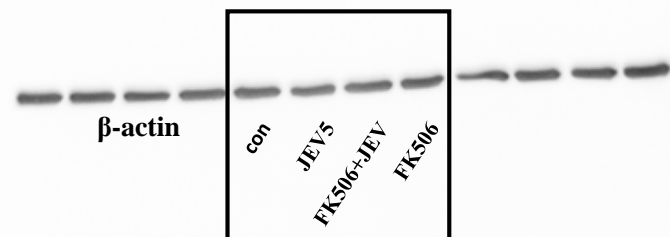

Supplement: Supplementary file 1 — Supplementary Material 1 [file 12868_2023_832_MOESM1_ESM.pdf]
